# A Review on Analytical Modeling for Collapse Mode Capacitive Micromachined Ultrasonic Transducer of the Collapse Voltage and the Static Membrane Deflections

**DOI:** 10.3390/mi12060714

**Published:** 2021-06-18

**Authors:** JiuJiang Wang, Xin Liu, YuanYu Yu, Yao Li, ChingHsiang Cheng, Shuang Zhang, PengUn Mak, MangI Vai, SioHang Pun

**Affiliations:** 1 College of Computer Science and AI, Neijiang Normal University, Neijiang 641100, China; tswangjade@gmail.com (J.W.); liyao2018@njtc.edu.cn (Y.L.); zhangshuanghua1@126.com (S.Z.); 2 State Key Laboratory of Analog and Mixed-Signal VLSI, University of Macau, Macau 999078, China; yb87445@connect.um.edu.mo (X.L.); fstmiv@um.edu.mo (M.V.); lodgepun@um.edu.mo (S.P.); 3 Department of Electrical and Computer Engineering, Faculty of Science and Technology, University of Macau, Macau 999078, China; 4 BeiDou and Wisdom Medical Doctor Workstation, Neijiang Normal University, Neijiang 641100, China; 5 School of Automotive Engineering, Wuhan University of Technology, Wuhan 430070, China; cmuts@yahoo.com

**Keywords:** analytical modeling, capacitive micromachined ultrasonic transducer (CMUT), collapse mode, collapse voltage, membrane deflection

## Abstract

Analytical modeling of capacitive micromachined ultrasonic transducer (CMUT) is one of the commonly used modeling methods and has the advantages of intuitive understanding of the physics of CMUTs and convergent when modeling of collapse mode CMUT. This review article summarizes analytical modeling of the collapse voltage and shows that the collapse voltage of a CMUT correlates with the effective gap height and the electrode area. There are analytical expressions for the collapse voltage. Modeling of the membrane deflections are characterized by governing equations from Timoshenko, von Kármán equations and the 2D plate equation, and solved by various methods such as Galerkin’s method and perturbation method. Analytical expressions from Timoshenko’s equation can be used for small deflections, while analytical expression from von Kármán equations can be used for both small and large deflections.

## 1. Introduction

Based on the electrostatic principle, the capacitive micromachined ultrasonic transducer (CMUT) has been one of the research trends for over the last two decades since its invention in the late 20th century. The CMUT can work in transmit mode to produce ultrasound, and can work in reception mode to receive ultrasound, as well. Therefore, it has been used in a variety of applications, such as nondestructive evaluation (NDE) [1,2,3], volumetric imaging [4,5], medical imaging [6,7,8], HIFU [9,10], gas sensor [11,12,13], hydrophone [14], pressure sensor [15,16,17], Doppler velocity measurement [18] and fingerprint sensing [19,20].

**Figure 1:**
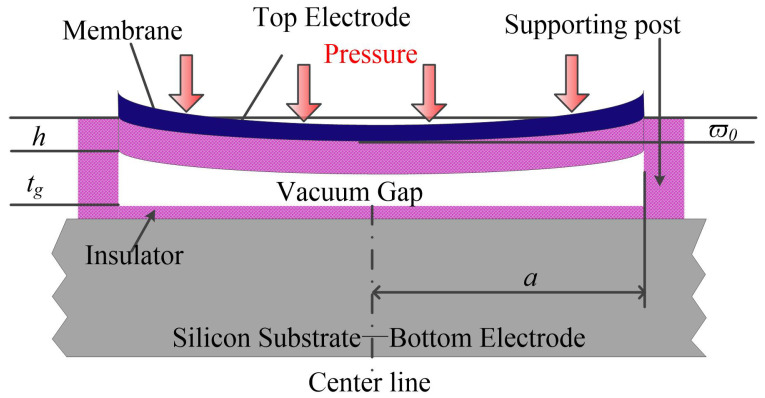
The cross-sectional view of a conventional CMUT with full top electrode. *a* is the radius of the membrane, *h* is the membrane thickness and $t_{g}$ is the vacuum gap height, $\varpi_{0}$ is the center deflection.

The main parts of a regular CMUT cell include a membrane together with a top electrode, a vacuum gap, one or two insulator layers, a supporting post connecting the membrane and the insulator layers and surrounding the vacuum gap as well, and a substrate that is highly doped and is used as a bottom electrode [21,22]. The membrane will be deflected toward the substrate due to the electrostatic force produced by a DC voltage applied between the two electrodes. Similarly, when there is a pressure applied on the membrane, the membrane will be deformed too. So, when the applied uniform pressure changes, the capacitance of the CMUT cell will change accordingly, as a consequence the CMUT can be used to detect the pressure [17,23]. The cross sectional view of a conventional CMUT is shown in Figure 1, in which the membrane will not contact the substrate during the working period, and the applied DC bias is less than the collapse voltage.

Compared with lead-zirconate-titanate (PZT) transducers, CMUT has a relatively small output impedance and this helps the CMUT to be used in air or in immersion applications, and be better coupled with the target [1]. The fractional bandwidth of CMUTs can be larger than 100% for immersion applications [1,21,24], which makes the CMUT suitable for ultrasound imaging. CMUT has many features, including wide bandwidth, low power consumption, easy to be integrated with front-end electronics, lack of self-heating, etc. [1,21,25,26].

Another type of micromachined ultrasonic transducer (MUT) is to use a layer of piezoelectric material as the vibrating part, and PZT and aluminum nitride (AlN) are the most commonly used piezoelectric materials [27]. CMUT works under a larger DC bias than PMUT, needs a smaller cavity, owns a larger bandwidth than PMUT [28], employs a more complicated fabrication process than PMUT [28,29], consumes more power than PMUT does [29].

One disadvantage of CMUTs is the relatively lower output acoustic pressure compared with that of PZT transducers. The loop gain of CMUT arrays in the pulse-echo experiment was 10 dB lower than that of PZT arrays [30]. An operation mode called collapse mode was introduced as a way to elevate the output pressure [31]. The electromechanical coupling efficiency $k_{t}^{2}$ was proved to be higher in collapse mode than in conventional mode [31]. Since then, two other operating modes have also been studied for CMUT to exploit its potency on more output pressure than the conventional mode. They are collapse snap-back mode and deep-collapse mode [25,32]. The main features of the operating modes are related to the different behaviors of the membrane under static working conditions. For collapse mode, the central part of the membrane is in contact with (collapses onto) the substrate [32,33]. As for collapse snap-back mode, the CMUT is first set in collapse state and then the membrane is allowed to lose contact with the substrate by adjusting the applied voltage, while the deep collapse mode refers to the state that the applied AC pulse magnitude is larger than the collapse voltage. All the three operating modes can enable the CMUT to output more pressure. This is especially suitable for imaging applications, as higher output pressure is preferable in such applications [25,34]. However, there exist some nonlinearities when a CMUT is working in deep-collapse or collapse snap-back modes [35].

**Figure 2:**
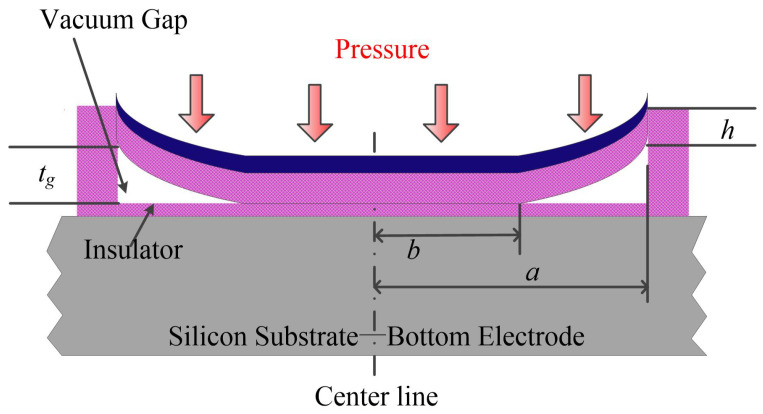
The cross-sectional view of a collapse CMUT with full top electrode. *b* is the collapse radius.

In general, a collapse mode CMUT can generate and detect ultrasound waves more efficiently than in conventional mode [25,32,33] and has a higher mechanical coupling coefficient [31]. Park et al. made a comparison of the conventional and collapse mode CMUTs both numerically and experimentally under the same DC bias, the devices under comparison had the same membrane thickness, material and peak frequency, and it was found that the output pressure of the collapse mode CMUT was about 2∼3.5 times of the conventional one [36]. The cross sectional view of a collapse mode CMUT cell is shown in Figure 2. A summary of advantages and disadvantages of collapse mode operation is in Table 1. Though the charging effect is more severe than in conventional mode, the charging effect can be decreased by alternating the polarity of the AC pulse [37,38]. The high second harmonic distortion (SHD) of collapse mode could decrease by shaping the input pulse [33].

The development of an accurate model for collapse mode CMUT is very important and necessary because of the lengthy and complicated development cycle of a CMUT and the subtle fabrication processes of CMUT [39,40]. Modeling of CMUTs is challenging since it involves multidisciplinary domains including electronics, acoustics, mechanics and their interactions. Three main types of modeling/simulation methods including finite element method (FEM), analytical modeling, and equivalent circuit modeling (ECM) have been developed to facilitate the design of CMUT devices prior to fabrication [41,42] for the sake of reducing design iterations. Brenner et al. presented a review on several aspects on CMUT including modeling, fabrication, and application, and both ECM and FEM modeling methods were discussed [26]. Eccardt et al. gave an overview of analytical modeling of CMUT and focused on the modeling of the conventional mode [43]. The analytical model has the advantages of intuitive understanding of the underlying mechanism and physical effects, and the resultant analytical solution can provide quick evaluation of designs for given specifications [44], it will not encounter non-convergent cases. The analytical models are powerful tools for CMUT design and fabrication. A governing equation is first constructed through understanding of the general physics of CMUT, adequate boundary conditions are then to be found to match certain applications, the final step is to solve the equations together with the boundary conditions by suitable mathematical methods.

The collapse voltage is a key factor to determine the working status of the CMUT because it determines the sensitivity, frequency response, and total acoustic output pressure [10,45]. Static membrane deflections can be used to derive the resonant frequency of a CMUT in vacuum, to derive the collapse voltage [43], to determine the electromechanical coupling coefficient, the transmitting intensity and the receiving sensitivity [45]. Many analytical related papers have been published for the development of CMUT theory and applications. The objective of this review is to highlight the related main methodologies and research techniques of CMUT modeling of the collapse voltage and the static membrane deflections, which will be presented in Section 2 and in Section 3, respectively. The summary will be in Section 4.

## 2. Collapse Voltage Models

Collapse voltage refers to the voltage that causes the membrane in contact with the substrate when the electrostatic force overcomes the membrane stiffening force. Bozkurt et al. showed that the collapse voltage $V_{collapse}$ of a CMUT cell is proportional to the $\frac{3}{2}$th power of membrane thickness under the uniform pressure assumption as in Equation (1) [46]. The expression was derived by using the plate-spring model, which regarded the membrane as a parallel plate suspended over the substrate through a spring. The pull-in voltage was decided as the voltage at which the derivative to the vertical displacement ∂V/∂x equaled to 0 where *V* was the applied DC voltage and *x* was the membrane displacement. They also pointed out that the application of that equation was limited because of the uniform pressure over the membrane surface assumption and a more general solution could come from computer simulation.

(1) $$V_{collapse}=\sqrt{\frac{8k{\left(t_{a}+\frac{\epsilon_{0}}{\epsilon }t_{m}\right)}^{3}}{27\epsilon_{0}A}}$$

where ϵ, $\epsilon_{0}$ were the permittivity of the membrane material and the permittivity of air, respectively, $t_{m}$ and $t_{a}$ were the membrane thickness, and the air gap height, respectively, *k* was the spring constant of the transducer membrane expressed as in Equation (2), *A* was the area of the membrane and also the the area of the top electrode due to full electrode design here.

(2) $$k=\frac{16\pi Y_{0}t_{n}^{3}}{\left(1-\rho^{2}\right)a^{2}}$$

where $Y_{0}$ was the Young’s modulus, ρ should be ν and ν was the Poisson’s ratio, $t_{n}$ was the thickness of the membrane, *a* was the membrane radius.

By assuming a CMUT cell as a parallel plate capacitor and using the similar principle, Yaralioglu et al. presented a detailed derivation of the collapse voltage as Equation (3), which is basically the same as Equation (1) [47].

(3) $$V_{collapse}=\sqrt{\frac{8k_{s}d_{eff}^{3}}{27A\epsilon_{0}}}$$

where $d_{eff}$ was the effective distance of dielectric materials, including vacuum gap, membrane and the insulator, *A* was the area of the top electrode, and $k_{s}$ was the spring constant of the moving electrode. It’s usage was as limited as the previous one.

**Figure 3:**
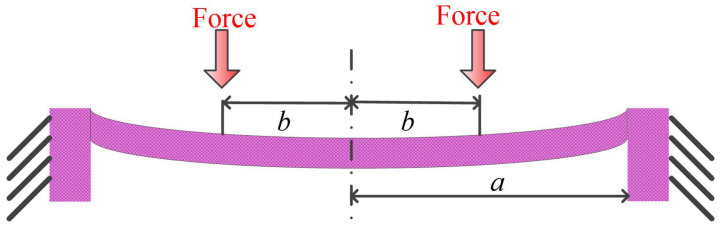
Circular clamped-edge plate concentrically loaded, a is the radius and b is the annular position.

Nikoozadeh et al. calculated the collapse voltage using an iteration method on the fixed boundary assumption and the linearity of the membrane deflections [48]. The membrane area coated with electrodes was divided into around 100 segments to make each annular ring segment small enough so that the electrostatic force could be regarded as uniform. The applied force and plate deflection were as shown in Figure 3, which is similar to page 63 in [49].

Closed form analytical solutions for annular membrane Equations (4) and (5) from Timoshenko [49] were used to calculate the membrane deflection due to the load on the small annular ring [48]. After that, superposition of all the deflections were used to simulate the whole membrane deflection. The displacement of the center of the membrane was used as a criterion to determine whether the solution converges or diverges. An iteration method was adapted to calculate the displacement under electrostatic force when the solution converged within an acceptable error tolerance. The collapse voltage was calculated using a binary search algorithm and a rapid diverge of membrane center displacement was used as the criterion. The method was compared with FEM and it was very efficient in calculation time while keeping an acceptable accuracy.

(4) $$\varpi_{i}=\frac{F_{i}}{8\pi D}\left[\frac{\left(a^{2}-r^{2}\right)\left(a^{2}+b_{i}^{2}\right)}{2a^{2}}+\left(b_{i}^{2}+r^{2}\right)\ln\frac{r}{a}\right],r>b_{i}$$

(5) $$\varpi_{i}=\frac{F_{i}}{8\pi D}\left[\frac{\left(a^{2}+r^{2}\right)\left(a^{2}-b_{i}^{2}\right)}{2a^{2}}+\left(b_{i}^{2}+r^{2}\right)\ln\frac{b_{i}}{a}\right],r\leq b_{i}$$

where *r* was the radial position, *a* was the membrane radius, $b_{i}$ was the inner radius of segmented annular ring, $F_{i}$ was the pressure on the *i*th segmented annular ring, and $\varpi_{i}$ was the membrane deflection due to $F_{i}$. *D* was the flexural rigidity of the membrane. The collapse voltage results matched well with FEM results, though there was no analytical expression of the collapse voltage.

Olcum et al. adjusted the collapse voltage to a more accurate one by adding a coefficient 0.7 to the previous Equations (1) and (3) as in Equation (6) [3].

(6) $$V_{col}≃0.7\sqrt{\frac{128\left(Y_{0}+T\right)t_{m}^{3}{\bar{t_{g}}}^{3}}{27\epsilon_{0}a^{4}}}$$

where *T* was the residual stress, and $\bar{t_{g}}$ was the effective gap height.

In their review paper, Eccardt et al. presented a method to measure the collapse voltage without collapsing the membranes by measuring the frequency shift at a known voltage [43] as shown in Equations (7) and (8). The $w_{rel}$ can be derived when $f_{Y}$ and $f_{R}$ were known from Equation (8) and the $V_{crit}$ can be solved from Equation (7)

(7) $$w_{rel}^{3}-2w_{rel}^{2}+w_{rel}-\frac{4}{27}{\left(\frac{V_{dc}}{V_{crit}}\right)}^{2}=0$$

(8) $${\left(\frac{f_{Y}}{f_{R}}\right)}^{2}=\frac{1-3w_{rel}}{1-w_{rel}}$$

where $w_{rel}$ was relative deflection and expressed as $w_{rel}=w_{dc}/h_{gap}$, $f_{Y}$ and $f_{R}$ were the frequency of maximum real conductance and the frequency of maximum real resistance, respectively. There needs to be extra work to get more accurate values of $f_{Y}$ and $f_{R}$ by measuring several times and obtaining the average value for each device.

Based on the assumption that the pressure on the membrane was uniform from the applied voltage, Wygant et al. [50] derived the shape function of a circular CMUT Equation (9) from Timoshenko’s plate theory, and further calculated the capacitance of the device by integration. On the basis of the capacitance and the principle of virtual work, the pull-in voltage (collapse voltage) was derived numerically.

(9) $$\varpi \left(r\right)=\frac{P_{0}a^{4}}{64D}{\left(1-\frac{r^{2}}{a^{2}}\right)}^{2}=\varpi_{pk}{\left(1-\frac{r^{2}}{a^{2}}\right)}^{2}$$

where $P_{0}$ was the pressure due to the atmosphere and the electrical force. $\varpi_{pk}$ was the membrane center deflection. The average membrane deflection $\varpi_{avg}$ was found to be one-third of the center deflection $\varpi_{pk}$, and the capacitance of the CMUT was derived by using $\varpi_{pk}$.

From Equation (9), a linear relationship between pressure $P_{0}$ and average displacement was derived in Equation (10). The linear spring constant $k_{1}$ could be expressed accordingly as Equation (10).

(10) $$\varpi_{avg}=\frac{P_{0}a^{4}}{192D}=\frac{F_{m}a^{2}}{192\pi D}=\frac{1}{k_{1}}F_{m}$$

where $F_{m}$ was the applied force, and $k_{1}$ was the linear spring constant. This linear constant was used for small deflection of membrane while another constant $k_{3}$ was derived for larger deflection of membrane, as well. By using the method in [51], the collapse voltage $V_{PI}$ was determined as Equation (11).

(11) $$V_{PI}=\sqrt{\frac{2\frac{dU_{m}\left(\varpi_{pi}\right)}{d\varpi_{avg}}}{\frac{dC(\varpi_{pi})}{d\varpi_{avg}}}}=\sqrt{\frac{2F_{m}\left(\varpi_{pi}\right)}{\frac{dC(\varpi_{pi})}{d\varpi_{avg}}}}$$

where $U_{m}$ was the mechanical energy, and *C* was the capacitance of the CMUT. The inconvenience of this method is the need of solving the pull-in voltage by numerical methods.

Ahmad et al. derived an analytical expression of collapse voltage as in Equation (12) from the pull in of MEMS device [52] using the classical thin plate theory under the assumption of small deflection of thin membrane. The solution used the Maclaurin’s theorem to simplify the expression of the electrostatic force on the membrane and the first two terms were used. The authors stated that the pull-in voltage was much larger when only the first term was used. A trial shape function (14) was used in the derivation process. They claimed that the pull in voltage predicted by their method was close to that from FEM results from other researchers.

(12) $$V_{PI}=\frac{5.46}{R^{2}}\sqrt{\frac{d_{0}^{3}D}{\epsilon_{0}}}$$

where $d_{0}$ was the initial gap height, $\epsilon_{0}$ was the vacuum dielectric permittivity, *R* was the membrane radius. The $d_{0}$ should be $d_{eff}$ that included the effect of the dielectric of membrane as shown in Equation (13).

(13) $$V_{PI}=\frac{5.46}{R^{2}}\sqrt{\frac{d_{eff}^{3}D}{\epsilon_{0}}}$$

(14) $$w\left(r\right)=a_{0}+a_{1}r+a_{2}r^{2}+a_{3}r^{3}+a_{4}r^{4}$$

where w(r) was the deflection profile, and $a_{0}$ to $a_{4}$ were coefficients to be determined.

Li et al. presented the pull-in voltage of the electrostatically actuated plate without other load or initial stress similar to Equation (12) [53] with the constant being 5.369 instead of 5.46. The trial function was in Equation (15), and actually was similar to Equation (12) with the exception that the effective gap height was used. So, Equation (13) can be regarded as one simple and effective expression of pull-in voltage.

(15) $$w\left(r\right)=\eta_{1}{\left(R^{2}-r^{2}\right)}^{2}$$

where $\eta_{1}$ was coefficient to be determined by solving the governing equation of the membrane deflection mathematically. The difference of the expansion of the electrostatic force between this and the previous method was that Taylor’s series was used to expand only one $\left(1-w/d_{0}\right)$ term in this paper while Maclaurin’s theorem was used to expand the ${\left[1-\left(w/d_{0}\right)\right]}^{2}$ in the previous paper. Both methods derived almost the same expression for the pull-in voltage. These would be more accurate because some nonlinear factors were taken into consideration.

Moreover, the expansion of Taylor’s series was extended into other areas, Li et al. used the Galerkin method to establish a reduced-order model for the collapse voltage and membrane deflection of CMUTs, considering the membrane being layered circular anisotropic microplates under DC bias voltage, residual stress and hydrostatic pressure [45]. The collapse voltage $V_{c}$ considering the residual stress and hydrostatic pressure was derived to be Equation (16).

(16) $$\begin{array}{ccc} V_{c} & = & \frac{d_{e}}{\sqrt{\epsilon_{0}}R^{2}}{\left\{504d_{e}D\prime +49.364d_{e}NR^{2}+2PR^{4}-Eqn\_A\right\}}^{1/2}\\ Eqn\_A & = & \sqrt{168d_{e}\left(8D\prime +0.818NR^{2}\right)\left(168d_{e}D\prime +16.091d_{e}NR^{2}+2PR^{4}\right)} \end{array}$$

where $d_{e}$ was the equivalent electrode distance, *P* was the hydrostatic pressure, *N* was the residual stress, *R* was the membrane radius, $\epsilon_{0}$ was the permittivity of the gap, and D′ was the effective stiffness of the multilayer plate that contained the stiffnesses of each layer.

A comparison of collapse voltage results from different analytical models is in Table 2.

The collapse voltage in Figure 7 of [47] was about 40 V, and, the collapse voltage in Table I of [48] was about 164 V from FEM, so the results from Equation (6) were closer to the data from the references. On the other hand, the results from Equation (13) were closer to Equation (6) than those from Equation (12).

## 3. Static Membrane Deflection Models

The membrane will be deformed under external force either coming from the electrostatic force due to voltage applied between the electrodes, or from the pressure on the membrane. The displacement of the membrane (together with the top electrode) can be classified into three categories according to the maximum displacement ($\varpi_{0}$) of the membrane as in Figure 1 to the thickness of the membrane (*h*) as follows [54], the range 0.2 to 0.3 was not listed separately, according to [55], that can be included in the nonlinear category:

- Small deflection, $\varpi_{0}$/h ≤ 0.2;
- Nonlinear deflection, 0.2 < $\varpi_{0}$/h < 1;
- Large deflection, $\varpi_{0}$/h ≈ 1.

For the conventional CMUT, the maximum displacement occurs at the center of the membrane, while for the collapse CMUT, the maximum displacement is equal to the gap height. From [42,48,54], thin plates refer to the membrane whose lateral dimensions *w* are in the range of 10 ≤ w/h ≤ 100 or at least an order more than the membrane thickness, and CMUTs fall into this category [47,48,52,56,57,58].

The classical Kirchhoff theory considers only the small deflection of the thin plate while the Mindlin theory takes into account of the first order shear force so it can handle the nonlinear and large deflection cases [59]. In [49], large deflection of plates were discussed, and lateral forces due to the radial and tangential elongation of the membrane were taken into consideration when deriving the governing equations of large membrane deformations. Some comparison results of elementary theory (without considering the elongation) and large deflection theory were demonstrated. For example, when the center deflection was a half of the membrane thickness, the result was 11 percent less than that of the method neglecting the stretching of the middle plane [49].

The deflection of CMUT membrane can be predicted by using the theory of plates [49]. Governing differential equations and boundary conditions for several shapes of plates and loading conditions were presented and solved. One case similar to the collapse of a CMUT membrane was presented where a circular plate was loaded with a uniform pressure and the center was supported by an absolutely rigid foundation and the boundary experienced a moment. The solution of membrane deflection under this combination of force and moment was proposed, which is similar to Equation (17).

(17) $$x\left(r\right)=C_{1}+C_{2}lnr+C_{3}r^{2}+C_{4}r^{2}lnr+\frac{r^{4}P}{64D}$$

where x(r) was the deflection profile, $C_{1}$ to $C_{4}$ were constants to be solved together with boundary conditions presented in the Appendix of [60]. Though the governing equations and boundary conditions were suitable for the small deflection of membrane, the collapse radii calculated in this paper using 200 V pulse was estimated to be over 10% difference between the FEM and circuit mode. Furthermore, one reason for that was the average force assumption when calculating the pressure [61].

**Figure 4:**
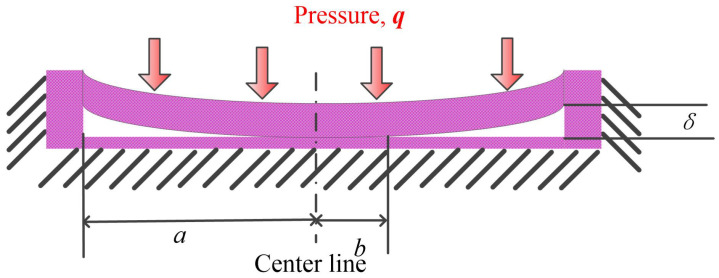
Deflection of circular plate with inner radius *b* (collapse radius).

Another case was similar and the inner radius (collapse radius) *b* was mentioned to be connected to the gap height δ and radius *a* and uniform pressure *q* as shown in Figure 4. The solution was not presented in the book.

Olcum et al. presented a nonlinear equivalent circuit model for a collapse mode CMUT and used the model to predict the collapse radius of a CMUT cell [60]. The small deflection of membrane was considered in this work for conventional and collapse mode, and uniform force distribution over the membrane surface was assumed. In the study, a full top electrode was used to increase the output pressure. The general deflection solution Equation (17) of uniformly distributed pressure from Timoshenko’s solution was used as the deformation expression. Its shortage was its inaccuracy due to the uniform force assumption [61].

In their later work, the accuracy was increased by using the exact electrical force distribution similar to Equation (18) and analytically calculating the collapsed membrane compliance and rms displacement-electrical force relationship [62].

(18) $$r\frac{d}{dr}\left(\frac{1}{r}\frac{d}{r}\left(r\frac{d}{dr}x\left(r\right)\right)\right)=\frac{1}{D}\int_{b}^{r}\left(P_{b}+\frac{\varepsilon_{0}V^{2}}{2{\left(t_{ge}-x\left(\xi \right)\right)}^{2}}\right)\xi d\xi$$

where x(r) was the bending profile of the membrane, P(r) was the circularly symmetrical pressure on a circular membrane, *D* was the flexural rigidity, $P_{b}$ was the static ambient pressure, $t_{ge}$ was the effective gap height, and *b* was the collapse radius.

The electrical force was approximated by a polynomial as in the right side of Equation (19). The deflection x(r) was calculated by an iterative routine, which was not presented in the paper. They performed simulations for conventional and collapse mode, both for small and large signal, and the results were consistent with the FEM (ANSYS, ANSYS, Inc., Canonsburg, PA, USA) results, with very faster simulation time and with 3 % difference in amplitude simulation.

(19) $$r\frac{d}{dr}\left(\frac{1}{r}\frac{d}{r}\left(r\frac{d}{dr}x\left(r\right)\right)\right)=\frac{1}{D}\int_{b}^{r}\left(P_{0}+\sum_{n}f_{n}r^{n}\right)rdr$$

where $P_{0}$ was the ambient pressure, and $f_{n}$ were the coefficients of the polynomials. The bending profile was not the main emphasis of that conference paper, so there was not enough information about the derivation process.

Similarly, Aydogdu et al. described the membrane deflection using the real force distribution instead of the equivalent uniform pressure under DC and AC voltage in the deflection equation from Timoshenko’s method Equation (18) [57,61]. By nondimensionalizing the parameters of *r*, *b* to $\bar{r}=r/a$ and $\bar{b}=b/a$, this approach deployed a K-th degree polynomial as in Equation (20) to get an analytical solution as in Equation (21) [57]. A detailed iterative routine was demonstrated to get all the constants ($A_{1}$ to $A_{4}$) and collapse radius *b* derived. This solution can predict the profile and the collapse point precisely and the solution can be used in collapse mode and collapse snap-back mode together with the conventional mode. The simulation results were claimed to be in good agreement with FEM results. They also made contributions to the simulation of collapsed CMUT arrays using a small signal model [63], based on the electrical force and energy relationship and using the Taylor expansion expression. Though the iterative process was used, the process gave an explicit explanation of the solving procedure as stated in [57].

(20) $$\bar{r}\frac{d}{d\bar{r}}\left(\frac{1}{\bar{r}}\frac{d}{\bar{r}}\left(\bar{r}\frac{d}{d\bar{r}}x\left(\bar{r}\right)\right)\right)=\frac{1}{D}\int_{\bar{b}}^{\bar{r}}\left(\sum_{n=1}^{K}c_{n}\xi^{n}\right)d\xi$$

where $c_{n}$ were coefficients of polynomials in fitting of pressure distribution.

(21) $$\bar{x}\left(\bar{r}\right)=A_{1}+A_{2}{\bar{r}}^{2}+A_{3}log\left(\bar{r}\right)+A_{4}{\left(\bar{r}\right)}^{2}log\left(\bar{r}\right)+\sum_{n=1}^{K}\frac{c_{n}{\left(\bar{r}\right)}^{n+3}}{{\left(n+1\right)}^{2}{\left(n+3\right)}^{2}}+{\bar{r}}^{2}\left(1-log\left(\bar{r}\right)\right)\sum_{n=1}^{K}\frac{c_{n}{\bar{b}}^{n+1}}{4(n+1)}$$

where $A_{1}$ to $A_{4}$ were constants to be solved, and *b* was the collapse radius. These constants and *b* were derived mathematically by a detailed iterative routine in [57].

Sénégond et al. discussed the non-linearity and collapse phenomenon of CMUT and developed a time-domain model able to predict the response of an immersed isolated CMUT and an immersed population of CMUTs, including the simulation of dynamic snap-back and collapse events, without any assumption on the loading effect of the fluid [35]. The membranes were assumed to be pistons when vibration and the deflections were small. The well-known 2D plate equation was used to determine the mechanical displacement of the diaphragm $w_{i}\left(x,y,t\right)$ as in Equation (22). The governing equation was then solved numerically based on the finite-difference theory. Though this solution was semi-analytical, it can provide information about the dynamic collapse voltage and showed that the maximum reachable dynamic collapse voltage was 1.8 times of the static collapse voltage.

(22) $$\nabla^{2}\left(D\nabla^{2}w_{i}\left(x,y,t\right)\right)-m{\ddot{w}}_{i}\left(x,y,t\right)=P_{self,i}\left(x,y,t\right)+P_{e,i}\left(x,y,t\right)+\sum_{j=1,j\neq i}^{N}P_{mut,ij}\left(x,y,t\right)$$

where *D* was the plate flexural rigidity, $P_{e,i}\left(x,y,t\right)$ was the distributed electrostatic force acting on the diaphragm, $P_{self,i}\left(x,y,t\right)$ was the self-pressure radiated in the fluid, *m* was the mass per unit area of the plate, and $P_{mut,ij}\left(x,y,t\right)$ was the mutual pressure between the CMUT indexed *i* and the CMUT indexed *j*.

Martin et al. developed an electro-mechanical, semi-analytic, reduced-order (RO) model of a fluid-loaded transmitting CMUT operated in collapse mode by approximating the deflection with a linear combination of six mode shapes. Membrane contact force with the substrate for collapse mode operation was included in the governing equation as in Equation (23) which was the non-dimensionalized version. The membrane deflection was small and the mid-plane of the membrane was assumed with no elongation, and the governing differential equation was solved using the Galerkin method. The results were compared with experimental results with a satisfactory accuracy [64].

(23) $$\frac{\partial^{2}\hat{w}\left(\hat{r},\hat{t}\right)}{\partial {\hat{t}}^{2}}+\nabla_{\hat{r}}^{4}\hat{w}\left(\hat{r},\hat{t}\right)=-\frac{{\hat{V}}^{2}\left(\hat{t}\right)}{2{\left(1+\hat{w}\left(\hat{r},\hat{t}\right)\right)}^{2}}+{\hat{H}}_{ab}erfc\left(\frac{\hat{t_{g}}+\hat{w}\left(\hat{r},\hat{t}\right)}{\hat{\sigma }}\right)+\frac{\hat{\tau }}{\hat{r}}\frac{\partial }{\partial \hat{r}}\left(\hat{r}\frac{\partial \hat{w}\left(\hat{r},\hat{t}\right)}{\partial \hat{r}}\right),0\leq \hat{r}\leq 1$$

where ${\hat{H}}_{ab}$ was the effective indentation hardness, erfc was the complementary error function, $\hat{\sigma }$ was the standard deviation of the combined surface roughness, and $\hat{\tau }$ was the residual stress parameter. This method took into consideration of the fluid-load effect and the membrane contact force with substrate, but there is no analytical expression of the deflection profile.

For the CMUT operation, a larger gap height means the possible maximum membrane displacement and permits more space for the membrane to vibrate when transmitting and can generate more output pressure, which is beneficial for imaging. In the nonlinear and the large deflection cases, the stretching of the mid-plane and further the lateral force must be taken into consideration [48,49]. Kupnik et al. mentioned that analytical method to solve large deflection under uniform load was complicated and Kármán equation can only be solved by successive approximation techniques [65]. Koymen included nonlinear behavior of a CMUT cell when it was driven by large amplitude signal, combined the parameters in the equivalent circuit model, which helped them to design CMUT without using FEM [42]. There was no analytical expression for collapse mode membrane deflection profiles prior to [57]. Von Kármán equations can be used to solve the nonlinear deflection of circular CMUT membrane in conventional mode [66]. Therefore, in 2016, Wang et al. proposed to advance the technique by introducing the perturbation method to solve the von Kármán equations so that a general solution can be reached. In the article, they demonstrated the technique and reached an analytical model for single layer collapse mode CMUT with large deflection membrane under a uniform pressure [67] with the fixed boundary condition at the rim of the membrane. The governing equations and boundary conditions were as Equations (24) to (26). The deflection profiles matched well with FEM results. The analytical expression was complicated, and the boundary condition was assumed to be fixed.

(24) $$r\frac{d}{dr}\frac{1}{r}\frac{d}{dr}\left(r^{2}N_{r}\right)=-\frac{1}{2}Eh{\left(\frac{d\varpi }{dr}\right)}^{2}$$

(25) $$Dr\frac{d}{dr}\frac{1}{r}\frac{d}{dr}\left(r\frac{d\varpi }{dr}\right)=rN_{r}\frac{d\varpi }{dr}+\frac{1}{2}q\left(r^{2}-b^{2}\right)$$

(26) $$\left.\begin{array}{c} \varpi =0\\ \frac{d\varpi }{dr}=0\\ r\frac{dN_{r}}{dr}+\left(1-\nu \right)N_{r}=0 \end{array}\right\}$$

where $N_{r}$ was the lateral force at *r*, ϖ was the deflection profile of the membrane, *h* was the membrane thickness, *D* was the flexural rigidity of the membrane, and *q* was the external uniform pressure.

To sum up, the analytical modeling of CMUT membrane deflections are shown in Table 3.

## 4. Summary

There are several methods to model collapse voltage of CMUTs with analytical expressions as a function of the effective gap height and the electrode area. An iteration method was used to calculate the electrostatic force and to determine the collapse voltage, and convergence of the displacement of the center of the membrane and the gradient of the applied voltage to that displacement were used as the criterion to find the collapse voltage. Another method adopted plate-piston-spring model to derive an analytical expression of collapse voltage and the derivative of voltage to vertical displacement was used as the criterion. Equation (6) or (13) would be more accurate for the collapse voltage.

Modeling of membrane deflections can be categorized into small, nonlinear and large. Currently, equations from Timoshenko, von Kármán equations and the 2D plate equation are used as the governing equations for both small and large deflection of membrane. Galerkin’s method and perturbation method are used to solve the governing equations. Analytical expressions from Equation (17) and Equation (18) can be used for small deflections, while analytical expression from von Kármán equations can be used for small and large deflections.

## Figures and Tables

**Table 1:** Advantages and disadvantages of collapse mode operation.

Parameters	Collapse Mode	References
Output pressure sensitivity	26.4 kPa/V, while 12.7 kPa/V for conventional	[33]
Receive sensitivity	higher than conventional	[33]
Mechanical coupling coefficient	higher than conventional	[31]
Center frequency	variable for collapse while fixed for conventional	[32]
Charging effect	more severe, but can be decreased	[37,38]
Second harmonic distortion	about 10 dB higher than conventional, but can be decreased	[33]

**Table 2:** Collapse voltages from different analytical models.

Method and Parameter Source	Equations (1) and (3)	Equation (6)	Equation (12)	Equation (13)
Parallel plate theory [47]	56.27 V	39.39 V	20.42 V	40.74 V
Semi-analytical algorithm [48]	218.75 V	153.13 V	124.16 V	158.35 V
Expansion of electrostatic force [52]	170.65 V	119.45 V	96.93 V	123.53 V
Expansion of electrostatic force [52]	2013.03	1409.12	326.75	1457.23

**Table 3:** Analytical modeling methods of membrane deflections of CMUTs in collapse mode.

Analytical Modeling Methods	Application Scenarios	Reference
Solution Equation (17)	For small deflections	[60]
Governing Equation (18) and its variations	For small deflections	[62]
2D plate equation	For small deflections, no analytical expression	[35]
von Kármán equations	For small and large deflections	[67]
